# Innovative IOP-Independent Neuroprotection and Neuroregeneration Strategies in the Pipeline for Glaucoma

**DOI:** 10.1155/2020/9329310

**Published:** 2020-04-10

**Authors:** James C. Tsai

**Affiliations:** Department of Ophthalmology, Icahn School of Medicine at Mount Sinai, New York Eye and Ear Infirmary of Mount Sinai, New York, NY, USA

## Abstract

While sustained reduction of intraocular pressure (IOP) has been shown to halt and/or delay the progressive death of retinal ganglion cells (RGCs) in glaucoma, there exists great interest in the development and validation of IOP-independent therapeutic strategies for neuroprotection and/or neuroregeneration. Multiple etiologies for RGC death have been implicated in glaucoma including defective axonal transport, ischemia, excitotoxicity, reactive oxygen species, trophic factor withdrawal, and loss of RGC electrical activity. However, IOP lowering with medical, laser, and surgical therapies is itself neuroprotective, and investigators are seeking to identify agents that are able to confer neuroprotection independent of IOP reduction, as well as providing for regeneration of nonviable RGCs and their axons to restore and/or maintain functional vision. These innovative strategies in the pipeline include investigation of neurotrophic factors, gene therapy, immune system modulation, and novel neuroregeneration pathways. Alongside this new knowledge, enhanced opportunities for discovery of vision preservation and/or restoration therapies must be weighed against the potential disadvantages of perturbing the complex central nervous system environment.

## 1. Introduction

Glaucoma, a multifactorial disease, is the second leading cause of blindness worldwide, affecting an estimated 76.0 million people worldwide by 2020 and increasing to 111.8 million by 2040 [1]. In addition, a high percentage of patients with glaucoma are unaware of their visual loss until permanent damage has occurred; for example, the percentage of undiagnosed patients in the United States ranges from 56% to 92% [2]. While glaucoma was historically defined as a disease of elevated intraocular pressure (IOP) greater than 21 mm Hg (the statistical upper 95^th^ percentile of IOP in normal subjects), population-based studies have shown that one-third or more of persons with open angle glaucoma (OAG) have normal levels of IOP [3]. Thus, the current definition of primary open angle glaucoma (POAG) is no longer contingent on the presence of “elevated IOP” but rather “a progressive, chronic optic neuropathy in adults where IOP and other currently unknown factors contribute to a characteristic acquired atrophy of the optic nerve and loss of retinal ganglion cells (RGCs)” [4].

Over the past several decades, multiple prospective studies have validated intraocular pressure (IOP) to be the most important risk factor for the development and progression of optic nerve damage in primary open angle glaucoma (POAG). The Ocular Hypertension Treatment Study (OHTS) demonstrated that the 5-year conversion rates of ocular hypertension to POAG were more than twice as high as for placebo-treated patients vs. medically treated patients (9.9% vs. 4.4%, respectively) [5]. Based on multivariate analysis, the Early Manifest Glaucoma Trial (EMGT) reported that the progression risk of early onset open angle glaucoma (OAG) was reduced by 50% with treatment, and the risk of glaucoma progression was decreased by 10% for each mm of Hg of initial IOP reduction [6]. In patients with OAG who present with normal IOP levels, the Collaborative Normal Tension Glaucoma Study (CNTGS) showed that only 12% of treated eyes had either progression of glaucomatous optic disc cupping or visual field loss compared with a 35% rate in control eyes (*p* < 0.0001) [7].

Currently accepted strategies for treatment of POAG, whether medical, laser, or incisional surgical modalities, aim to lower IOP below a presumed threshold level [4, 8, 9]. Though these IOP-lowering therapies have been proven to slow and/or halt progression of the glaucomatous damage and therefore are neuroprotective in nature, slow progress has been made in developing IOP-independent neuroprotection and/or neuroregeneration strategies [10, 11]. While neuroprotection strategies to enhance the ability of target cells in the central nervous system (CNS) to withstand pathological insults have shown substantive promise in animal models, none have been reported to be clinically effective in human clinical trials [11]. In addition, early stage intervention to prevent disease development and/or progression would likely require evolution of improved algorithms and technologies to enable and/or enhance this earlier detection. Novel experimental strategies are exploring disease modification/intervention to prevent against optic nerve head (ONH) damage and/or retinal ganglion cell (RGC) death without depending on IOP lowering. One upstream goal would target intervention at an early stage of the glaucomatous disease process to halt or slow down the underlying neurodegenerative process. Various etiologies for RGC death have been implicated including defective axonal transport, ischemia, excitotoxicity, reactive oxygen species, trophic factor withdrawal, and loss of RGC electrical activity [12, 13]. Technological advances allowing for in-vivo imaging of RGCs may assist in clear identification of dying RGCs, providing opportunities to evaluate interventions with appropriate clinical applications. For example, human clinical trials are now underway utilizing a noninvasive real-time imaging technique with the fluorescent biomarker annexin A5 to detect in-vivo rates of apoptosis in RGCs [14].

Though IOP lowering with conventional therapies is itself neuroprotective, the development of non-IOP dependent neuroprotective therapies is definitely possible. The overarching goal would be to intervene with these agents at the earliest stage of the disease and/or its progression. In addition, regeneration of nonviable RGCs and their axons would be highly desirable to restore and maintain functional vision. However, randomized clinical trials investigating oral memantine and topical brimonidine have not demonstrated a clear neuroprotection benefit in patients with glaucoma [11, 15]. For the latter pharmacologic agent, patients with low-pressure glaucoma had slightly less visual field progression with topical administration of the alpha2-adrenergic agonist brimonidine tartrate 0.2% compared to the beta-adrenergic antagonist timolol maleate 0.5% [15]. However, significantly more brimonidine-treated patients (28.3%) discontinued study participation due to drug-related adverse events as compared to the timolol-treated patients (11.4%) [15].

### 1.1. Neurotrophic Factors

Neurotrophic factors have shown promise in retarding progression of neurodegenerative diseases. Active preclinical and clinical studies are ongoing investigating ciliary neurotrophic growth factor (CNTF), brain-derived neurotrophic factor (BDNF), glial cell line-derived neurotropic factor (GDNF), and others [16, 17]. Exogenous application of BDNF to the retina and viral vector has been shown to induce amplification of BDNF expression in retinal neurons and to be effective in neuroprotection of RGCs [17]. One study showed that in an axotomy-induced cell death model in the adult C57BL/6J mouse, GDNF and CNTF potently and synergistically rescued RGCs when compared to control retinas up to 8 weeks after the lesion [18].

Human CNTF was investigated in a phase 1 clinical safety trial with delivery of cells (designated NTC-201 and derived originally from the human retinal pigment epithelium cell line ARPE-19) transfected with the human CNTF gene and insulated within surgically implanted capsules [19]. The study enrolled 10 participants who received vitreous implanted CNTF encapsulated devices that were semipermeable to allow the neurotrophic factor to reach the retina in therapeutic levels for retinal degeneration. The study conclusion was that CNTF is safe for human retina and may have future applications for a wide range of retinal degenerative diseases including glaucoma [19]. In contrast, BDNF cannot successfully cross the blood-brain barrier, and thus, it is not surprising that there are no studies to date reporting BDNF's therapeutic effects for retinal degeneration [16]. Erythropoietin (EPO), a naturally occurring cytokine used to treat anemia by inhibiting apoptosis in erythrocyte progenitors, has been shown to be neuroprotective [20–22]. Experimental studies have reported that intravitreal injections of EPO rescue RGCs and prevent caspase-3 activation in axotomized rats (Sprague-Dawley), as well as retard against RGC loss in a rat model of ocular hypertension [20, 21]. In the DBA/2J mouse model of glaucoma, EPO was shown to promote RGC survival without affecting the IOP [23]. Furthermore, EPO-induced neuroprotection has been demonstrated to follow a bell-shaped dose-response curve in vitro and in vivo [20]. Additional studies have reported that one-time intravitreal administration of EPO (at doses up to 625 ng) does not cause adverse effects on retinal vasculature, retinal anatomy, or retinal function as assessed by electroretinography (ERG) in Sprague-Dawley rats and New Zealand white rabbits [24, 25].

### 1.2. Gene Therapy

Another approach to prevent and/or retard RGC loss is via gene therapy to deliver antiapoptotic neurotrophic proteins [16]. As noted above, a human Phase 1 safety trial demonstrated the successful delivery of CNTF by cells transfected with the human gene CNTF gene [19]. In brown Norway rat RGCs, the gene for Bcl-XL (amplified from C57BL/6J mouse whole-brain cDNA), a prosurvival and antiapoptotic protein, has been successfully delivered via adeno-associated virus (AAV) and HIV-Tat-derived fusion proteins [26]. In addition, an AAV vector (AAV-BDNF-WPRE) capable of efficient transfection of Wistar rat RGCs has been developed to deliver BDNF [27]. In the study, rats that had overexpression of the AAV-BDNF gene were more resistant to RGC death in an hypertensive model of glaucoma. To counteract the reduction of BDNF effect over time as a result of downregulation of tropomyosin-related receptor-B (TrkB) receptors, a novel AAV2 gene therapy vector has been designed to code both the BDNF ligand and the TrkB receptor and delivered via intravitreal injection to C57BL/6 mice [28].

There has been great interest in understanding Wallerian degeneration of axons and synapses, an active process that is intricately associated with RGC death as described in C57/BL6J mice [29]. The slow Wallerian degeneration (Wld(s)) gene specifically delays axonal and synaptic degeneration in multiple neurodegenerative conditions. Since altered mitochondrial responses to degenerative stimuli likely play an important role in the neuroprotective Wld(s) phenotype, targeting proteins involved in this phenotype may lead to novel therapies in glaucoma as noted in Wld^S^ mice [30, 31]. In addition, the neuroprotective effects of the Wld(s) gene are correlated with proteasome expression rather than inhibition of apoptosis [32].

An alternative to viral vector delivered gene therapy is the use of stem cells to transfer these specific genes. One study reported the feasibility of mesenchymal stem cell-based delivery of BDNF gene to Sprague-Dawley rat retina [33]. Following subretinal injections of rat bone marrow mesenchymal stem cells administered to axotomized rat retina, significant expression of BDNF was observed for 4 weeks following transplantation of these stem cells [33]. A more recent publication reported the development of CD-1 mouse multipotent retinal stem cell (MRSC)-derived RGCs that express key RGC characteristics with the potential for neuroprotection and regeneration of damaged RGCs [34]. In this study, three-dimensional (3D) cocultures were used to validate the model for transfection efficiency and BDNF bioactivity measurements [34].

### 1.3. Immune System Modulation

Tumor necrosis factor-alpha (TNF-*α*) has been shown to cause a cascade of events that lead to loss of RGCs. In a C57BL/6 mouse model of glaucoma, increased levels of TNF-*α* were demonstrated to cause microglial activation, loss of oligodendrocytes in the optic nerve, and loss of RGCs in an irradiation-induced murine model of ocular hypertension (OHT) [35]. In contrast, no increased death of RGCs above baseline was observed when animals were treated with TNF-*α* inhibitors, and/or knockout mice unable to produce TNF-*α* were studied [35]. Based on their experimental results, the investigators concluded that blocking TNF-*α* signaling or inflammation may be someday proven helpful in treating glaucoma. Based on the above findings, future treatments for glaucoma may involve modulation of the TNF-*α* pathway including direct blockage of TNF-*α* function and inhibition of downstream microglial activation [35]. These various possibilities include blocking antibodies that interfere with TNF-*α*, soluble receptor(s), and a TNF-*α*-converting enzyme inhibitor(s). A more recent study reported that antagonism of the TNF-*α* signaling pathway delays axotomy-induced RGC loss in a C57Bl/6 mouse model of traumatic neuropathy though the effect was not as favorable as observed with activation of survival pathways by BDNF [36]. Furthermore, combination treatment with BDNF and the small cell permeable molecule (R7050) that inhibited TNF-*α*/TNF receptor 1 (TNFR1) did not demonstrate superiority to BDNF alone and did not improve RGC survival [36].

In the near future, the method of T-cell-based vaccination for morphological and functional neuroprotection may be possible since this therapeutic option has been shown to be effective in retarding RGC cell loss in an inbred Lewis and Sprague-Dawley rat model of glaucoma [37]. In experimental animals with chronically elevated IOP, vaccination with the synthetic copolymer glatiramer acetate (Cop-1) was shown to be protective against IOP-induced loss of RGCs by eliciting a systemic T-cell-mediated response capable of cross-reacting with self-antigens in the eye [38]. However, no benefit was observed when Cop-1 was administered in inbred Lewis and Sprague-Dawley rats deprived of T-cells, thus supporting the hypothesis that the effect was T-cell mediated [38]. A recent interim report of a randomized placebo-controlled double-masked clinical trial in 38 patients with primary angle closure glaucoma failed to show any difference in visual field progression (or RNFL thickness change) between the Cop-1 and placebo groups [39]. However, there was slight improvement of the mean deviation (MD) at week 16 in the Cop-1 patient group compared to worsening of MD in the placebo group [39]. While promising, exploration of these T-cell-mediated immune response pathways should be equalized with the potential risk(s) of inducing autoimmune disease.

## 2. Neuroregeneration

In the adult mammalian CNS, the growth of injured axons is very limited following pathologic insult. For patients with glaucomatous neuropathy, the eventual goal for therapy will likely be one of visual restoration (partial and/or complete). Theoretically, this approach would be possible through neuroregeneration, in which there is reversal of the process of RGC death through regeneration of functional axons and restoration and/or recovery of the appropriate visual input. In experimental animal models, a variety of methods have been developed to deliver stem cells to replace RGCs and their axons, with the goal of ultimately re-establishing functional vision [40, 41]. To date, however, no IOP-independent neuroprotection and/or visual function enhancement trials have been successfully conducted in humans affected with glaucoma [41].

A recently described scientific protocol in rodents provides a novel and cost-effective means to differentiate human embryonic stem cells (hESCs) into RGC-like neurons, thus broadening the scope for future cell replacement therapy in glaucoma [36, 42]. In this protocol, human RGC-like cells were observed to migrate successfully into the rat ganglion cell layer approximately one week following cellular transplantation via intravitreal injection [42]. In Sprague-Dawley rat eyes with unilateral optic nerve crush injury, topical administration of a pharmacologic inhibitor of Rho-associated protein kinase (ROCK) and norepinephrine transporter (Net) has been shown to promote RGC survival and regeneration [43]. While the ROCK/Net inhibitor (AR-13324) lowered IOP as expected, the investigators observed RGC survival and optic nerve axonal regeneration at significantly higher levels compared with placebo [43].

Another therapeutic strategy for neuroregeneration in glaucoma involves modulation of axonal outgrowth in the CNS. A novel approach involves neuroprotection and neurorestoration via inhibition of the myelin-associated Nogo receptor pathway. Three CNS myelin proteins, Nogo-A, Nogo-B, and Nogo-C, stimulate the Nogo receptor, thereby inhibiting neurite outgrowth by causing growth cones to collapse through activation of ROCK [44]. The use of anti-Nogo antibodies has been shown to upregulate CNS regeneration, as well as improve sensory and motor function in both rats and primates with spinal cord injuries [44]. In spinal-injured Sprague-Dawley rats, administration of the soluble function-blocking NgR ectodomain (NgR(310)ecto-Fc protein) causes axonal sprouting with subsequent correlation to improved spinal cord electrical conduction and improved locomotion [45]. Soluble LINGO-1 (LINGO-1-Fc), acting as an antagonist of these axonal outgrowth inhibition pathways by blocking LINGO-1 binding to NgR1, significantly improves functional recovery and promotes axonal sprouting after spinal cord injury in Sprague-Dawley rats [46]. In Sprague-Dawley rat glaucoma models, a human NgR1 blocking protein (NgR1(310)-Fc) injected intravitreally promoted RGC axonal regeneration following optic nerve crush injury, as well as demonstrated a neuroprotective effect in a microbead glaucoma model [47].

Changes in Krüppel-like transcription factors (KLFs) are known to be associated with decreases in intrinsic axon growth capacity during development of adult mammalian CNS neurons. KLFs are also involved in regulating axon growth in CNS neurons including RGCs. Investigators have reported that knockdown of KLF9 via short hairpin RNA (shRNA) promotes long-distance optic nerve regeneration in adult Sprague-Dawley rats and mice of both sexes [48]. Moreover, a novel physiologic role for the interaction of KLF9 and JNK3 (c-Jun N-terminal kinase 3) has been discovered to be critical for KLF9 axon-growth-suppressive activity [48].

## 3. Conclusion

Recent discoveries of the molecular mechanisms underlying POAG have provided new insights into the disease's complex pathogenesis. This enhanced understanding offers opportunities for the development of novel sight preserving and/or vision restoration therapeutic strategies for this blinding disease. Moreover, the refinement of animal models of glaucoma that more closely mimic the pathophysiology of the human disease will assist in facilitating development of these novel strategies. In addition, the restoration of vision after de novo genesis of photoreceptors in congenitally blind mice (Gnat1^rd17^ Gnat2^cpfl3^ double mutant mice) offers promise that a similar regenerative process could be achieved for RGCs [49]. Finally, any exploration of these novel pathways for neuroprotection and neuroregeneration should be balanced and weighed against the real risk(s) of disrupting and damaging the complex CNS milieu in vivo (e.g., induction of autoimmune disease with manipulation of the T-cell-mediated immune response).
